# Systematic review of wearables assessing medication effect on motor function and symptoms in Parkinson’s disease

**DOI:** 10.1038/s41531-025-00943-y

**Published:** 2025-05-22

**Authors:** Emma Packer, Héloïse Debelle, Harry G. B. Bailey, Rana Zia Ur Rehman, Alison J. Yarnall, Lynn Rochester, Lisa Alcock, Silvia Del Din

**Affiliations:** 1 Translational and Clinical Research Institute, Faculty of Medical Sciences, Newcastle University, Newcastle upon Tyne, United Kingdom; 2 Research Institute for Sport and Exercise Sciences, Liverpool John Moores University, Liverpool, United Kingdom; 3 Johnson & Johnson Innovative Medicine, 50-100 Holmers Farm Way, High Wycombe, Buckinghamshire HP12 UK; 4 National Institute for Health and Care Research (NIHR) Newcastle Biomedical Research Centre (BRC), based at Newcastle upon Tyne Hospitals NHS Foundation Trust, Newcastle University and the Cumbria, Northumberland and Tyne and Wear (CNTW) NHS Foundation Trust, Newcastle upon Tyne, UK; 5 The Newcastle Upon Tyne Hospitals NHS Foundation Trust, Newcastle upon Tyne, UK

**Keywords:** Parkinson's disease, Parkinson's disease

## Abstract

To improve motor function and symptoms, people with Parkinson’s (PwP) typically take dopaminergic medication. In PwP, wearable technology (WT) can provide objective insights into medication effect. This review aims to identify and explore literature which uses WT to quantify the effect of medication on motor function and symptoms in PwP (PROSPERO 2022 CRD42022310018). Nine databases were searched between January 2000-October 2024. Downs and Black quality appraisal assessed study quality. PRISMA guidelines were followed. Amongst the seventy-nine included studies, 50 different WTs were placed across 20 locations on the body, and medication effect was monitored on 13 different motor functions/symptoms. There was great heterogeneity amongst protocols, but many studies were performed in controlled environments, exploring short-term medication effects (ON vs OFF). Medication effect varied, improving certain variables, and having no effect on others. Future research should identify gold-standard protocols to explore medication effect in real-world settings, over prolonged periods.

Registration and Protocol PROSPERO 2022 CRD42022310018.

## Introduction

Parkinson’s disease (PD) primarily influences motor function (e.g., gait), with cardinal motor symptoms of bradykinesia, tremor, rigidity, and postural instability^1,2^ reducing an individual’s quality of life^3,4^. As the prevalence of PD is rapidly increasing, becoming one of the leading causes of disability worldwide, significant burden is being placed on healthcare services^5^. Therefore, improving motor function and symptoms in people with PD (PwP) is of utmost importance.

At present, there are no disease modifying treatments for PD^6^, with individuals predominantly relying on dopaminergic medication (such as levodopa) to manage impaired motor function and symptoms^3,7^. Due to the progressive nature of PD, medication complexity increases with disease progression, with adherence to prescribed dosage and timing essential to alleviate motor symptoms and improve function. However, in PwP increased medication complexity is associated with suboptimal medication adherence, poor medication responses, and motor fluctuations (ON-OFF periods), with prolonged levodopa usage often inducing involuntary movements, termed dyskinesia^8–10^. Clinicians typically adapt medication regimens based on short, infrequent appointments, in which patients attend during specific medication stages (ON-OFF).

Current research exploring medication effect on motor function and symptoms in PwP typically relies on comparing scores from the Movement Disorder Society—Unified Parkinson’s Disease Rating Scale (MDS-UPDRS)^11^ between ON-OFF medication states. However, in PwP motor symptoms frequently fluctuate throughout the day, therefore one-off subjective measures, such as the MDS-UPDRS^12,13^ may not adequately reflect this. Wearable technology (WT), such as body-worn devices, may overcome these limitations, and have a transformative effect on individual’s care^14^. This technology has the potential to provide a more accurate reflection of an individual’s symptomology and lead to more optimal medication regimen adaptation. In line with the National Health Service (NHS) vision to ‘use the most advanced technology for patients’, NICE have recommended five wearable devices for remote monitoring^15^. For example, the wrist-worn Parkinson’s KinetiGraph (PKG®, Global Kinetics Corp, Australia) continuously monitors tremor, bradykinesia, and dyskinesia^16^, and sends medication reminders. Technology such as this can provide useful insights for objectively exploring medication effect in PwP.

Current research which uses WT has highlighted the positive impact of medication on motor function and symptoms in PwP, such as gait speed^17^, tremor^18^, and bradykinesia^19^. However, there is great heterogeneity within this research, with medication effect generally explored in controlled environments, over short time periods. To optimise medication regimens in PwP and enhance clinical management, a clear understanding of current research and its limitations is paramount.

This review aims to identify and explore literature which uses WT to quantify the effect of medication on motor function and symptoms in PwP. Objectives were to report: (i) the WT used and placement on the body; (ii) the assessed motor function and symptoms; (iii) the effect of medication on motor function and symptoms and the protocols used to explore this; and (iv) to provide evidence-based recommendations for future research.

## Results

### Search Yield

**Fig. 1:**
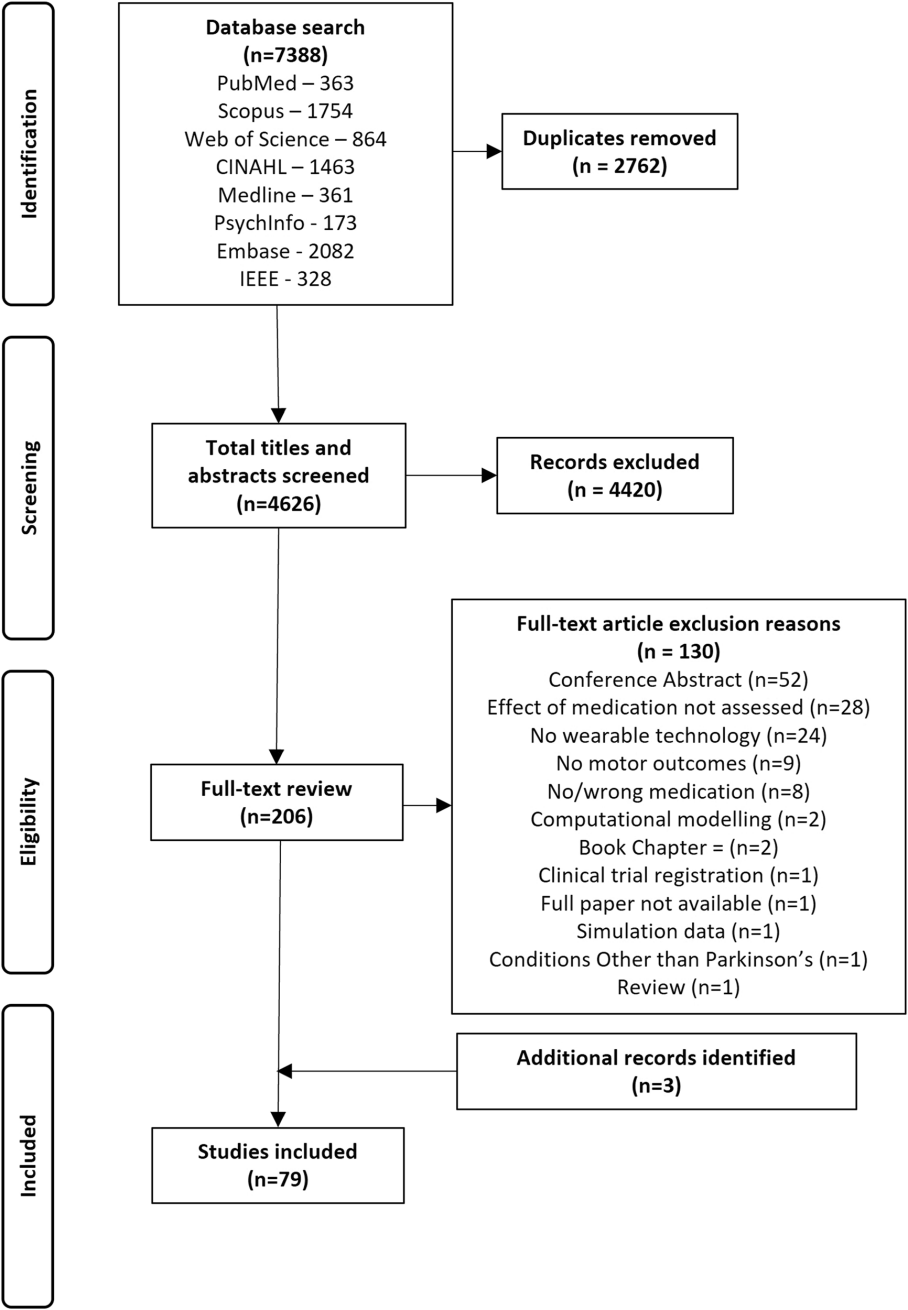
PRISMA flow diagram.

The search yielded 7388 papers (Fig. 1). After 2762 duplicates were removed, 4626 papers were screened by title and abstract. A total of 4420 papers met the exclusion criteria, and 209 papers underwent full-text review (three additional papers were identified through reviewing reference lists in included papers). From the full-text review of the 209 papers, 130 were excluded. The final sample comprised 79 articles. Reasons for exclusion are provided in Fig. 1. A summary of all included studies is presented in Supplementary Table 5.

### Study Characteristics

Of the 79 articles, the quality appraisal identified one ‘excellent’ article, 63 were rated ‘good’, 14 were rated ‘moderate’, and one was rated ‘poor’ (Supplementary Table 4). The majority of studies were observational (73/79; 92%), with six interventional studies. Studies were conducted across 19 countries. The majority were conducted in the United States of America (*n* = 25), followed by Italy (*n* = 11), Germany (*n* = 9), Sweden (*n* = 7), Spain (*n* = 5), Portugal (*n* = 5), The Netherlands (*n* = 5), Israel (*n* = 4), and England (*n* = 3). Two studies were conducted in each of the following countries: Greece, Ireland, China, Japan, and Canada. A single study was conducted in each of the following countries: Argentina, Australia, Belgium, Denmark, and South Korea. Three studies were performed across multiple countries. Studies were conducted between 2004 and 2024, with the majority published after 2017 (57/79; 72%) (Supplementary Table 5).

### Wearable Technology

Across the included studies, fifty different WTs were used to monitor motor function and/or symptoms (Supplementary Table 6). The most commonly used WT was an Opal device (*n* = 10)^20–29^, followed by The Physilog (*n* = 6)^30–35^, the Parkinson’s Kinetigraph (PKG) (*n* = 6)^36–41^, and the Shimmer 3 (*n* = 3)^40,42,43^. Eight studies utilised a smartphone^31,35,44–48^ or an iPad^49^, which monitored motor function and/or symptoms using embedded accelerometers. Shoe insoles were used in two studies^50,51^, and one study used smart gloves (iTex)^52^. Three studies^53–55^ did not describe the make or model of the technology used, generally stating that an IMU was used, and the company which developed it. Three studies simply stated that either tri-axial accelerometers or gyroscopes were utilised^56–58^, but provided no further detail, with one paper stating a motion sensor was used^59^. 63/79 studies utilised validated technology (Supplementary Table 4), with the remaining studies not providing enough information to identify if the technology or algorithms were validated.

Across the included studies a variety of sensors were used to monitor motor function and/or symptoms (Supplementary Table 5) (only sensors used to monitor function/symptoms are reported e.g., if the WT contained two sensors, but only data from one sensor was used the other will not be reported). Specifically, 30 studies used accelerometers; 4 studies used gyroscopes^54,58,60,61^; 31 studies used both accelerometers and gyroscopes; 7 studies used a combination of accelerometers, gyroscopes, and magnetometers^22,23,25,28,62–64^; and 3 studies used a combination of accelerometers, gyroscopes, magnetometers, and barometers^31–33^. One study used flexion sensors^52^, and three studies did not specify which sensors were used^29,45,65^.

### Wearable Technology Placement

**Fig. 2:**
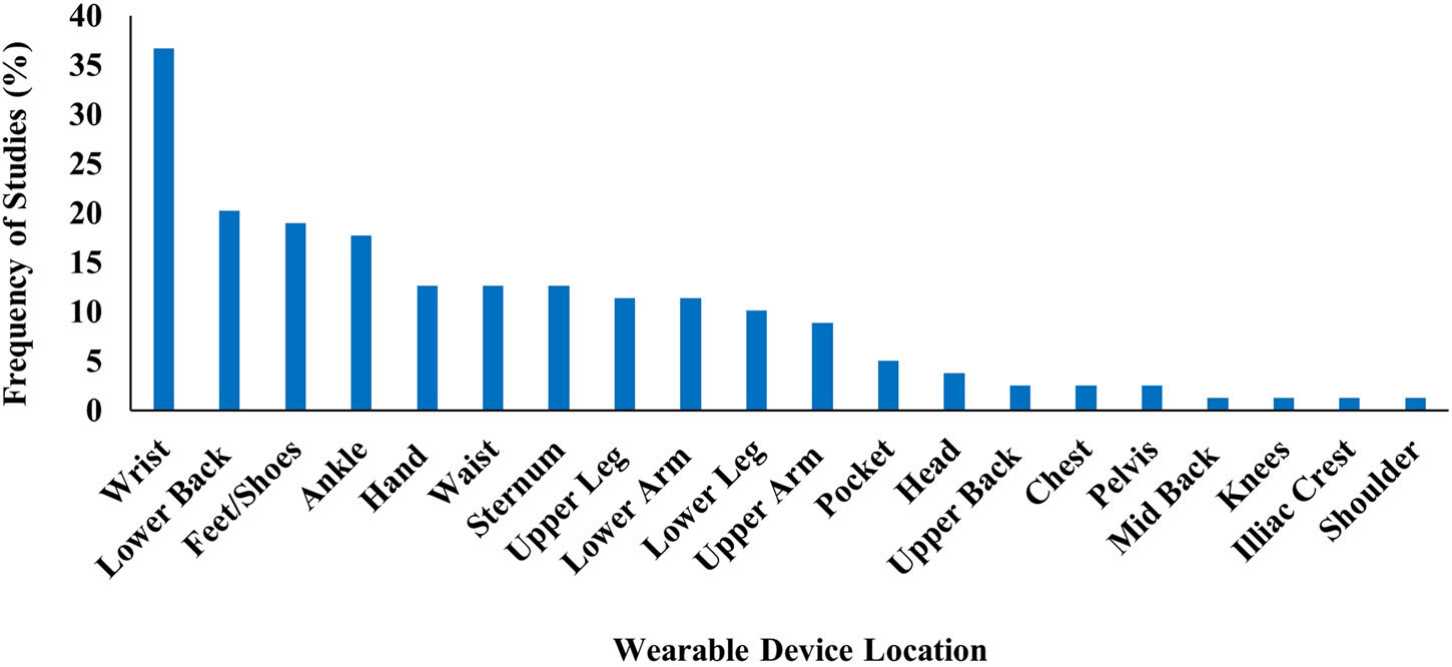
Proportion of studies, which place wearable technology at different locations on the body.

All but three studies^45,48,65^ reported on the placement of WT on the body. Across the included studies, WT was placed across 20 locations on the body (Fig. 2 and Table 1). The wrist was the most common (*n* = 29), followed by the lower back (*n* = 16), feet/shoes (*n* = 15), ankle (*n* = 14), hand (*n* = 10), waist (*n* = 10), sternum (*n* = 10), and upper leg (*n* = 9)^19,62,64,66–71^. Smartphones were placed in trouser pockets in 4 studies^31,35,44,46^, and on the waist in one study^47^, with two studies^45,48^ not specifying the location of phone placement. Pastorino et al.^56^ and Rahimi et al.^62^ provided limited information on WT placement, simply stating that technology were worn on the legs and arms, without specifying which section of the limb.

A single piece of WT was worn in 33 studies (Table 1). Sixteen studies placed technology on the most affected limb, this included the most affected wrist (*n* = 15)^25,34,36–39,42,54,60,61,68,72–75^, ankle (*n* = 4)^54,58,60,61^, hand, lower arm, and lower leg (*n* = 1)^76^. Technology was worn bilaterally (in addition to other locations) in 38/79 studies. This included both feet/shoes (*n* = 15)^19,20,23,29,30,32,33,50,51,55,64,67,69,77,78^, wrists (*n* = 14)^20,21,23,24,28,31,35,40,43,63,79–82^, upper legs (*n* = 8)^19,62,64,67–71^, upper arms (*n* = 7)^19,62,64,67–69,71^, lower arms (*n* = 8)^19,62,64,67,69–71,83^, ankles (*n* = 9)^21,22,24,28,31,35,43,59,63^, hands (*n* = 7)^19,52,62,64,67,69,84^, lower legs (*n* = 6)^19,62,64,67,69,84^, shoulders (*n* = 1)^69^, and the knees (*n* = 1)^57^ (Table 1).

**Table 1:**
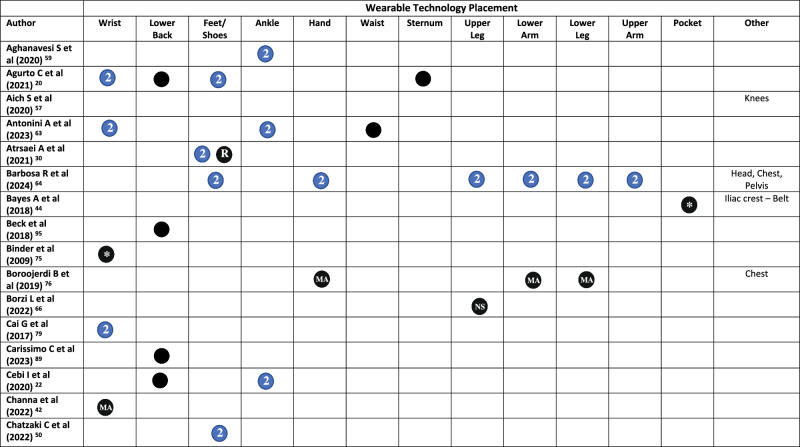

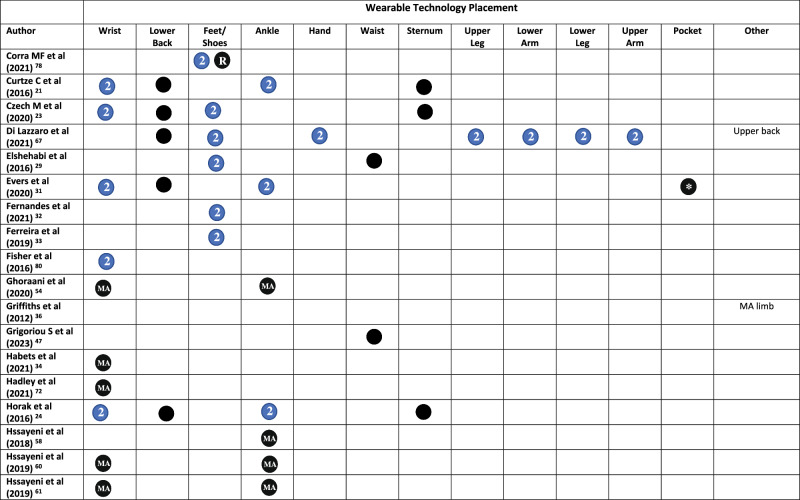

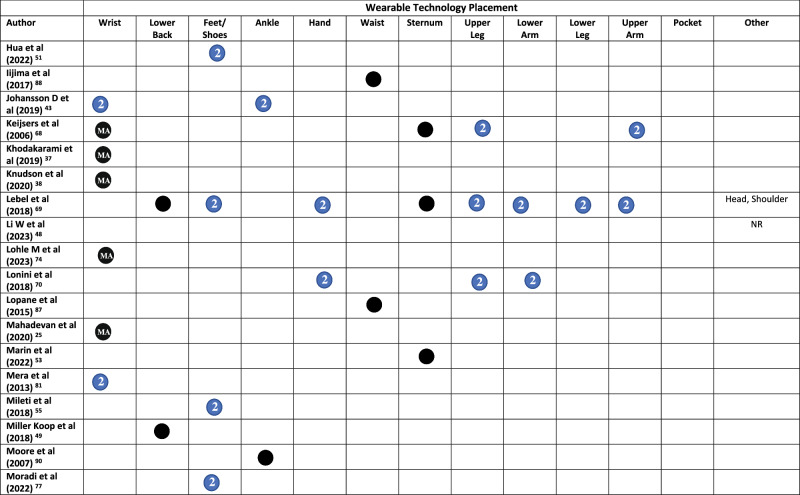

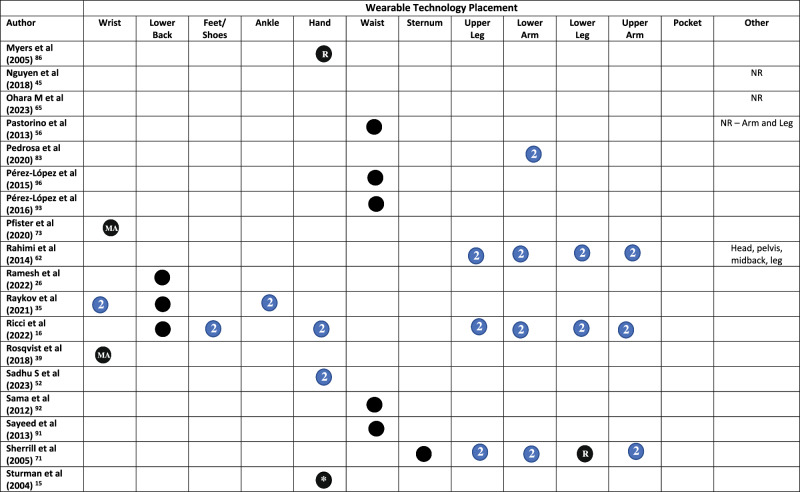

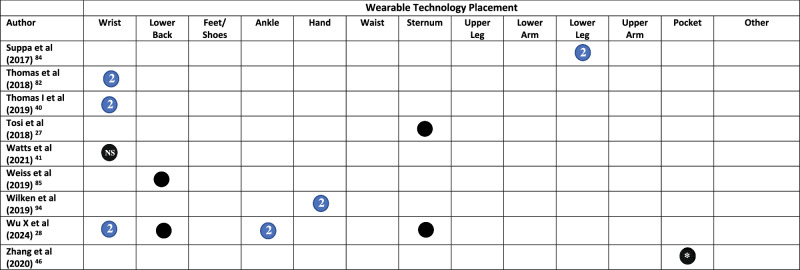
Wearable technology placement on the body in each study

Symbol: Black Circle = Unilateral/Single, Blue Circle with a number 2 in = bilateral (left and right), * = optional, *MA* Most Affected, *D* Dominant, *ND* Non-dominant, *NR* Not Reported, *R* Right.

### Motor Function and Symptoms

**Fig. 3:**
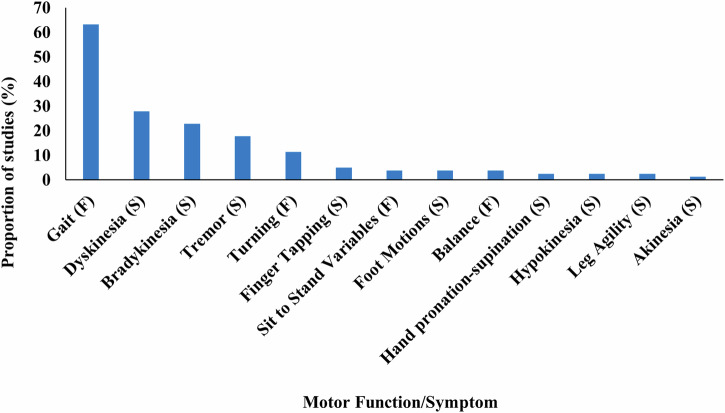
Frequency of studies, which assessed each motor function/symptom. (F) = Motor Function, and (S) = Motor Symptoms.

Across the included studies, 13 motor functions/symptoms were monitored using WT (Fig. 3) (Supplementary Table 5). A variety of protocols were used to assess each motor function/symptom, with Supplementary Table 5 summarising the protocol used to assess each motor function/symptom in their respective study.

Across the included studies, four motor functions were assessed: including gait, turning, sit-to-stand, and balance. The most commonly assessed function was gait, monitored in 50/79 studies. Specific gait measures included: gait speed, step or stride length and time, swing time, asymmetry and variability of gait variables, average step number, average distance walked, cadence, mean gait cycle duration, anticipatory postural adjustments (APAs), and freezing of gait. Turning (e.g., number of steps to perform a turn, timed-up-and-go variables, turn duration, and turn velocity) was assessed in 9/79 studies^19,21,28,29,49,64,67,69,85^. Sit-to-stand (STS) variables (e.g., number of attempts, peak and mean speed, and trunk acceleration) were assessed in 3/79 studies^27,43,53^, and balance (e.g., postural sway) was assessed in 3/79 studies^21,24,45^ (Supplementary Table 5).

Across the included studies, six motor symptoms were assessed, including dyskinesia, bradykinesia, tremor, foot motions, hypokinesia, and akinesia, with outputs from finger tapping, hand pronation-supination tasks, and leg agility also assessed. Dyskinesia was monitored in 22/79 studies, with the majority of studies detecting the presence and/or duration of dyskinesia and detecting how long after medication intake dyskinesia was present. Bradykinesia presence and/or severity was assessed in 18/79 studies. Tremor was assessed in 14/79 studies, with specific tremor metrics including tremor amplitude, consistency, and severity. Finger tapping regularity and variability was monitored in 2/79 studies^76,86^. Participants performed finger tapping activities in an additional study^43,87^, but the output was not directly monitored, instead using the finger tapping movements to detect dyskinesia presence. Specific foot motions (toe and heel tapping) were monitored in three studies^51,67,76^. Hypokinesia^68,88^, hand pronation-supination^43,76^, and leg agility^67,76^ were monitored in two studies. Akinesia^56^, was monitored in one study.

### WT Placement and Motor Function/Symptoms

**Fig. 4:**
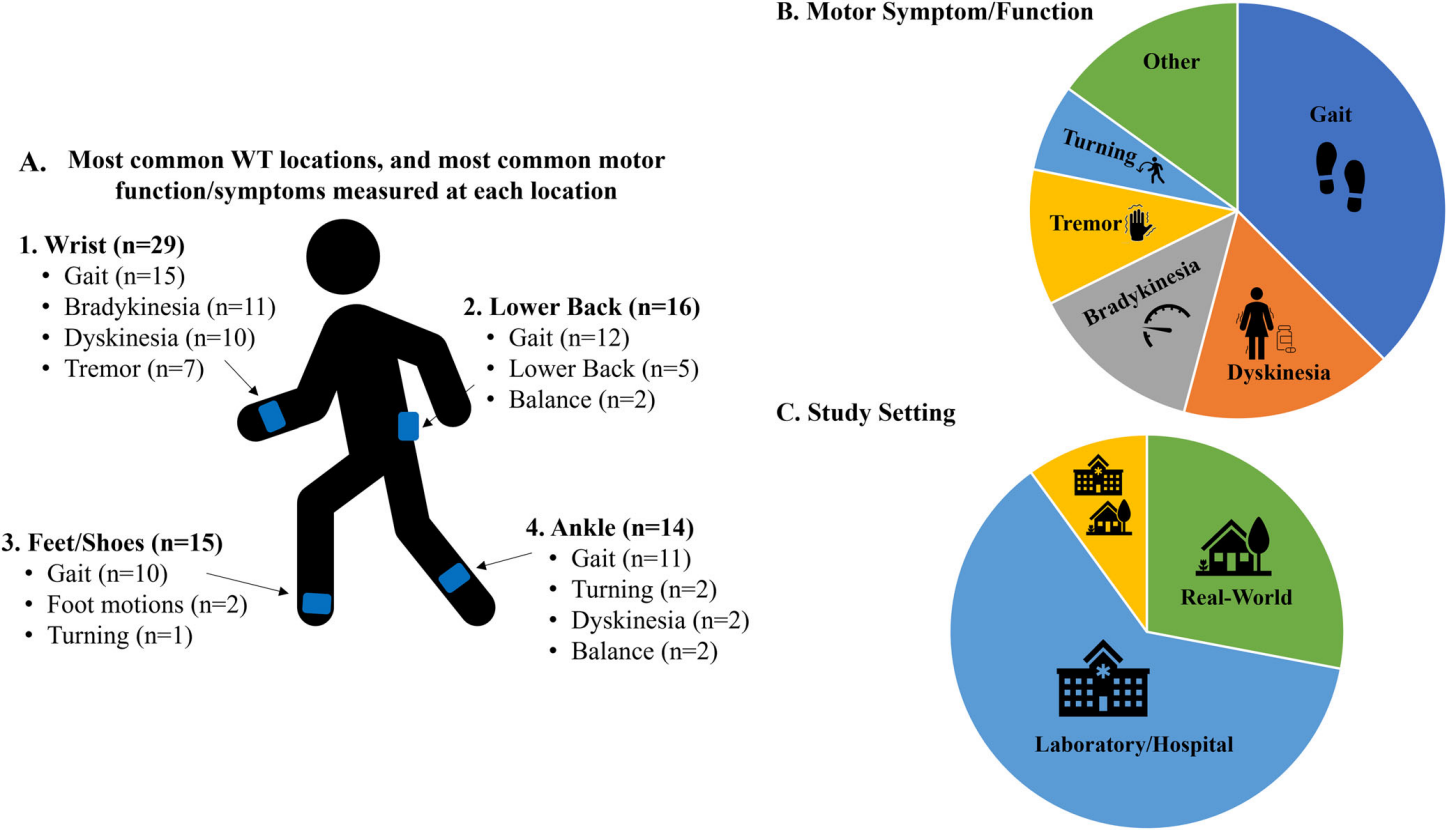
Summary of key findings. **A** Most common wearable technology (WT) locations, and the most common motor function/symptom monitored at that location. **B** The percentage of studies which monitored the five most common motor function/symptoms. **C** The percentage of studies monitored in each study setting. (Made in PowerPoint, with previously created icons).

To monitor each motor function/symptom, WT was placed across various locations on the body (Fig. 4). Specifically, to monitor gait, WT was placed across 17 different locations, but was most commonly placed on the wrist (*n* = 15) (Table 1 and Supplementary Table 5). Simiarly, to monitor dyskinesia, WT was placed across six locations, and most commonly placed on the wrist (*n* = 10)^38,40,41,43,63,72,74,80–82^. To measure bradykinesia and tremor, WT was placed across seven locations each, but most commonly on the wrist in eleven and seven studies, respectively. To monitor turning, WT was placed across 10 locations, but most commonly on the lower back (*n* = 5)^21,28,49,67,85^. Of the three studies to monitor finger tapping, WT was placed on the hand in two studies^76,86^, and wrist^43^ and lower arm^76^ in one study each. To monitor hand pronation-supination WT was placed on the wrist^43^ and hand^76^ in one study each. Two studies monitored balance, with both placing WT on the sternum, wrist, lower back, and ankle^21,24^, with the third study not reporting WT placement. In the two studies, which monitored hypokinesia, WT was placed across five different locations, including the waist in one study^88^ and the wrist, sternum, upper leg, and upper arm in another study. Akinesia was monitored in one study, with WT placed on the arms, legs, and waist^56^. Sit to stand was monitored at the sternum (*n* = 2)^27,53^ and wrist (*n* = 1)^43^. To detect foot motions WT was placed on the feet/shoes in 2 studies^51,67^. To monitor leg agility, WT was placed on the upper^67^ and lower^76^ legs in one study each.

### Monitoring Protocol

Forty-nine studies (62%) were conducted in controlled settings (e.g., clinic, hospital, or laboratory), 22/79 studies (28%) were conducted in the real-world (e.g., home and community), and 8/79 studies (10%) were conducted in controlled settings and in the real-world (Supplementary Table 5).

Fifty-eight studies monitored motor function and/or symptoms during specific ON-OFF medication states. Six studies monitored during ON-OFF states and continuously, over 6.5–8.5 h (depending on PD severity)^73^, 12-hours ^30,78^, 2-days^42^, 3-days^79^, 6-days^40^, and 7-days^80^. Nine studies performed only continuous monitoring over 24-hours^88^, 2-days^74^, 3-days^44^, 7-days^38,56,89^, 10-days^36^, 14-days^77^, and 42-days^75^.

Three studies recruited de novo PwP, assessed their motor function and/or symptoms, and monitored motor function and/or symptoms after being on Parkinsonian medication for 3 and 6 months^65^; 12 months^67^, or 6 months and 12 months^19^. One study monitored participants for three days prior to a clinician adapting their medication routine and monitored them again for 3-days during weeks 3 and 5 after the medication change^72^. Another study monitored participants for 6-days prior to and after a clinician adapted their medication regimen^41^. One study monitored independent participants in the ON-state, and the OFF-state^51^.

### ON-OFF Monitoring

Amongst the 64 studies which monitored motor function and/or symptoms in specific ON-OFF states, there was large heterogeneity in the timings used for ON-OFF assessment, with 22 variations for ON-state and 16 for OFF-state (Table 2). Table 2 summarises the different timings used for ON-OFF monitoring across the 64 studies, with timings written as reported in their respective papers.

ON-state was most commonly assessed one hour after medication intake (21/79 studies), and OFF-state was most commonly assessed following a 12-hour medication withdrawal (27/29 studies) (Supplementary Table 5 and Table 2). Seven studies did not define the time period for OFF-state monitoring^27,51,53,58,63,90,91^ and 13 studies did not define timings for ON-state monitoring^27,31,42,49,51,53,58,63,71,80,90–92^, simply stating that participants were monitored before and after medication intake; in ON-OFF medication states; or defined that they were monitored in 30 minute intervals after intake, but did not stipulate when the recording finished. One study monitored participants for up to 4-hours after medication intake but did not define the time periods for ON-OFF recording^87^ (Table 2). Six studies provided little information for OFF-state timing, simply stating the assessments were done “Early”^50^, in the “Morning”^86,93^, the “First dose of the day not taken”^71,92^, or that assessments were performed “immediately before and just after medication”^48^.

Although monitoring over a prolonged time period, certain studies only compared before and after medication intake within set timings, for example Moradi et al. monitored over 14-Days but only used data from 30, 45, 60, and 75 min before and after medication intake^77^.

To assess medication effect, 12 studies administered suprathreshold doses of participant’s normal medication dose. This included administering 120%^40,43^, 125%^21,24^, 150%^22,32,33,59,64,82,83^, and between 150 and 50%^47^ of the normal dose. In one study, if participants did not switch between medication states, an extra medication dose was taken, or levodopa was withdrawn^68^, and in another study all participants were given 250/25 mg of levodopa/carbidopa^94^. Additionally, to assess medication effect, Rosqvist et al. gave all participants 200 mg tablet of Madopark Quick, dissolved in water^39^. Another study administered a suprathreshold dose (150%) to induce dyskinesia^59^.

**Table 2:** Timings for motor function/symptoms monitoring before and after medication intake

ON-State (*n*=number of studies)	OFF-State (*n*=number of studies)
1 h after medication intake (*n* = 21)	12 h medication withdrawal (*n* = 27)
Every 0.5h until ON state confirmed or 90 min after medication intake (*n* = 1)^25^	10 h medication withdrawal (*n* = 2)^69,82^
45 min after medication intake (*n* = 2)^37,69^	Overnight withdrawal (*n* = 5)^29,31,34,60,61^
50 min after medication intake (*n* = 1)^18^	No medication after 10 pm (*n* = 3)^47,80,81^
Clinician reported ON-state (*n* = 3)^60,61,73^	0.5-1 hr and 1 hr before next dose (*n* = 1)^25^
Participant reported ON-state (*n* = 5)^26,34,54,85,96^	50 min before next dose (*n* = 1)^59^
Participant ‘feeling at their best’ (*n* = 1)^45^	3 h after medication intake (*n* = 1)^23^
20, 40,60, 80 min after medication^64^	Participant reported OFF-state (*n* = 2)^26,96^
30, 60, 45, 75 min window before and after medication (*n* = 1)^77^	Clinician reported OFF-state (*n* = 1)^73^
Every 30 mins for up to 150-min after intake (*n* = 1)^70^	First dose of the day not taken (*n* = 2)^71,92^
Up to 230-min after intake (*n* = 1)^43^	Morning (*n* = 2)^86,93^
Up to 280 min after intake (*n* = 1)^59^	Early (*n* = 1)^50^
Up to 320 min after intake (*n* = 1)^82^	24 h withdrawal from all medication except L-DOPA; no L-DOPA after 10pm; no amantadine for 1 week^47^
Continuously for 3 h after intake (*n* = 1)^68^	Withdrawal from Dopamine Agonists for 72 h and off anitparkinsonian medication for 12 h^28^
Once every hour for 3 h (*n* = 1)^81^	Withdrawal from Dopamine Agonists for 24 h and 12 h from anitparkinsonian medication^94^
2 h after medication intake^28^	1 hr before next dose^79^
Between 1-2 h after medication in lab; 1-1.5 h in real-world ^78^	
30-120 min after medication^29^	
Every 30 min after medication for 5 h^47^	
Every 20 min until the lowest UPDRS-III score was monitored (*n* = 1)^94^	
1-2, 2-3, 3-4, 4+ hours after medication^52^	
Every 15 min for 90 min, then every 30 min for up to 4 h (*n* = 1)^87^	

### Medication Effect on Motor Function and Symptoms

Only 36/79 studies (45%) reported findings in a clinically relevant manner—i.e., they reported the strength and direction of medication effect on clinically measurable motor functions and/or symptoms, e.g., reporting the value/change in step length following medication intake. Across the included studies various terminology was used for medication, including ‘dopaminergic’, ‘parkinsonian medication’, ‘antiparkinsonian’, ‘levodopa’, and ‘medication’. Therefore, to avoid misinterpreting results terminology used in this section, follows that reported in the respective study manuscripts.

#### Gait

Studies generally observed an improvement in gait following medication intake. Specifically, following ‘dopaminergic’ medication intake, gait speed^22,29,50,78,95^, stride length^21,22,33,50,84,90^, and step length^50^ increased. Similarly, following ‘levodopa’ intake, gait speed^21,24,33,39,64,84^, step and stride length^64^, walking distance^79^, the average number of steps over an hour^79^, and the size of APAs during step initiation increased^21^, and FOG duration decreased^84,93^. Additionally, stride length increased following ‘antiparkinsonian’ medication^28^. One study found that following 6-months of levodopa medication (de novo at baseline), sequence effect (progressive decrement of movement amplitude and speed) of stride length was ameliorated, in comparison to intravenous levodopa effects on stride length^65^.

Across the included studies, opposing medication effects were also reported for gait. For example, following medication intake, mean gait cycle time signifantly decreased in one study^21^, but stayed the same in another^22^; step count to complete a 7 m walking trial^39^ significantly decreased following medication intake, but stayed the same for a 20 m walking trial^29^; and swing time significantly increased following ‘levodopa’ intake^64^, but stayed the same in another study^21^. Four studies explored the effect of ‘levodopa’ on cadence^21,49,64,84^, with only one study observing a significant improvement in cadence (in participants with severe PD - 109 steps in OFF state, and 112 steps in ON state)^21^. However, following the addition of selegiline, a monoamine oxidase inhibitor, cadence also improved^88^.

No studies observed a statistically significant change in stride time^31,49,84^, stride time variability^95^, or swing time asymmetry^22^ following ‘dopaminergic’ medication. Similarly, one reported that ‘levodopa’ had no statistically significant effect on the variability and asymmetry of step length and time; the asymmetry of stance and swing time; double support variability, or stride time variability^64^. Furthermore, following ‘levodopa’ intake, no effect was identified on dynamic stability metrics during gait, specifically double support time^21^, step/stride time^64^, and stance time^21^.

Atrsaei et al. compared in clinic and real-world gait speed, and found that in the real world 24/27 participants performed strides with a faster gait speed than those measured in clinic (termed exceptional strides), and these exceptional strides mostly occurred in the ON state, three hours after medication intake^30^. Parkinson’s severity and FoG influenced medication effect in various studies. For example, in one study, mean step time only improved in participants with severe PD (Hoehn and Yahr III and IV)^21^, with another observing that ‘levodopa’ predominantly increased step velocity and stride length in those with FoG, but not in those without^84^.

#### Turning

Studies which monitored turning, generally observed an improvement in turning variables following medication intake. Specifically, Di Lazzaro compared turning variables during the Timed Up and Go test (TUG) when participants were de novo and 6 and 12 months after they were prescribed ‘dopaminergic’ medication, and observed that the number of steps, and turn duration significantly reduced, and turning velocity increased^67^. This was further supported by Ricci et al., who found that ‘levodopa’ improved the time to complete the TUG test, with reductions in turn duration, and turn to sit time^19^. Miller Koop et al. also reported that following ‘dopaminergic’ medication, participant’s turn velocity increased and turn duration reduced^49^. Similarly, one study observed that during turning, the peak velocity of the shank was significantly higher following ‘antiparkinsonian’ medication intake^28^.

However, three studies did not identify any significant changes in turning following ‘dopaminergic’ medication intake, specifically no increase in turn duration^29^, velocity^69^, or changes in turn strategy, with participants always performing the ‘overlapping’ strategy, where part of turning and sitting are completed concurrently^85^. However, Elshehabi et al. who observed no significant improvement in turn duration following medication intake, observed a significant increase in turning peak velocity following medication intake^29^. Similar to gait, one study observed that PD severity and dyskinesia influenced the effect of medication on turning. Specifically, Curtze et al. observed that medication only reduced turn duration in the most severe participants (Hoehn and Yahr III and IV), and turn velocity only increased in participants with dyskinesia^21^.

#### Dynamic and Static Balance

Two studies reported a negative effect of ‘levodopa’ on various balance measures^21,24^. Specifically, Curtze et al. reported that following ‘levodopa’ intake participants were less stable, with sway velocity increasing during an instrumented gait test (measure of dynamic stability)^21^. Horak et al. also observed that ‘levodopa’ worsened (increased) sway area, and improved (decreased) sway frequency and trunk stability during gait, with sway frequency and trunk stability moving closer to that of healthy controls^24^. Further negative impacts of ‘levodopa’ were identified on mediolateral and anterior-posterior postural sway velocity, which increased following ‘levodopa’ intake and variability during quiet standing, but only in those with Hoehn and Yahr III and IV^21^. The third study to monitor balance did not report the effect of medication in a ‘clinically relevant’ manner, instead reporting on the accuracy of algorithms to classify participant’s medication state^45^.

#### Sit to Stand Outcomes

Two studies observed no effect of ‘dopaminergic’ medication on STS variables^27,49^, with one study reporting an ‘improvement’ in STS performance in 5/7 participants, but providing no further detail^53^. Whereas, Miller Koop et al. found no difference in time taken to perform STS following ‘dopaminergic’ medication^49^, and Tosi et al. observed no statistically significant differences in mean speed of STS following ‘dopaminergic’ medication^27^.

#### Dyskinesia

Across the included studies, following ‘dopaminergic’ medication intake, dyskinesia was induced in 3/19 participants^43^, and participants spent statistically more time with dyskinesia^93^. Additionally, Curtze et al. identified that the presence of dyskinesia influenced other motor symptoms, with dyskinetic participants swaying significantly more following ‘levodopa’ intake^21^. Furthermore, Grigoriou et al. monitored trunk hyperkinesia as a measure of dyskinesia and found that following ‘levodopa’ intake, trunk hyperkinesia peaked after 90 min, whereas following ‘levodopa’ and ‘ropinirole’ trunk hyperkinesia peaked after 60 min, and decreased more quickly than just levodopa alone^47^. Many studies which monitored dyskinesia, assessed the accuracy of their algorithms to detect its presence in ON-OFF medication states, without reporting how dyskinesia differed between medication states.

#### Bradykinesia

Across the included studies, bradykinesia-related variables generally improved following dopaminergic medication^19^. Specifically, following ‘dopaminergic’ medication intake, less time was spent with bradykinesia^93^, movement amplitude increased during leg agility tasks, and rapid alternating hand movements also increased. Furthermore, toe tapping^67^, and wrist pronation speed^71^ also increased following ‘levodopa’ intake, with one study observing the largest change in PKG bradykinesia scores, 30 min after medication intake^38^.

#### Tremor

Included studies generally reported improvement in tremor-related measures following medication intake. Specifically, tremor amplitude^18,94^, consistency/regularity^18,25^, and time spent with a tremor^93^ were all reduced following ‘dopaminergic’ medication intake. Additionally, Binder et al. observed that following the addition of cabergoline (a dopaminergic agonist, no longer recommended for PD) for 6 weeks, tremor duration and amplitude significantly reduced^75^. However, Ricci et al. observed that none of their tremor features significantly improved following ‘levodopa’ intake^19^. Similarly, Di Lazzaro observed no significant improvement in sensor-measured tremor metrics (power and amplitude during MDS-UPDRS 3.15 and 3.17) within 6, 12, and 36 months of beginning ‘dopaminergic’ medication^67^.

#### Finger Tapping

Following ‘dopaminergic’ medication intake, finger tapping amplitude regularity and variability increased^86^. Sadhu et al. also found 1-2 h after ‘medication’ intake, dominant finger tapping frequency was significantly higher^52^.

#### Other Motor Symptoms

Iijima et al. found that the addition of 4 mg of Selegiline to participant’s medication regimens did not significantly improve hypokinesia, but it did increase the amplitude and range of forces for some participants, and motor fluctuations in 12/14 participants disappeared^88^.

Boroojerdi et al. produced an aggregated score from sensor derived activities (e.g., gait, pronation-supination, tremor, toe tapping, leg agility), and compared this aggregated score before and after medication for each participant^76^, therefore, medication effect on individual motor function and symptoms cannot be obtained, but ‘mean in-clinic scores were similar before and after levodopa intake’^76^. Hadley et al. reported that 11/14 participants experienced a significant change in one motor symptom (tremor, bradykinesia, and dyskinesia) following medication intake, but did not stipulate which motor symptom specifically improved, or its statistical significance^72^. Studies, which assessed akinesia^56^, leg agility and hand pronation-supination^76^ did not report clinically relevant insights, instead reporting on the accuracy of algorithms to detect ON-OFF states.

#### Other outcomes

All studies included in this review monitored PwP before and after medication intake, and therefore had the ability to report clinically relevant insights into medication effect on motor function/symptoms in PwP. However, as the main aim of many studies was to report on the accuracy of their algorithms to detect symptoms, or detect ON-OFF state, many studies did not report the motor function/symptom values or direction of medication effect. For example, one study monitored toe and heel tapping, in ON-OFF medication states, to identify if they could be used to distinguish medication states^51^, and another developed an algorithm for ON/OFF detection, based on bradykinetic gait and choreic dyskinesia^96^. Twenty-four studies reported on the accuracy and precision of their algorithms to detect or distinguish between ON-OFF states, with one study reporting on the accuracy to detect if the participant had taken their medication or needed to take it^45^. Similarly, one study reported on the ability of sensor measured gait scores to discriminate ON-OFF medication states^23^. Other studies explored which algorithm could best detect differences in ON-OFF medication state using STS variables^27,53^. Other studies reported on the accuracy of algorithms to detect the presence of bradykinesia^42,70^ or dyskinesia^70,73,80,81^, tremor severity^42^, and gait phase estimation in ON-OFF states^55^. One study assessed the ability of sensor measured dyskinesia to reflect clinically measured dyskinesia continuously over 10 days^36^. Another study investigated whether sensor measured motor symptoms correlated with a treatment response objective index (TRIS) (a model of participant’s response to levodopa intake)^43^. Other studies reported changes in non-clinical (signal-based) measures, such as spectral power, acceleration magnitude, root mean square of acceleration^34^, the spectral arc length of the Fourier magnitude spectrum^95^, and power spectral density^31^.

### Participant Feedback

Only eight of the 79 studies assessed either usability, wearability, tolerance, and/or feasibility of WT. Specifically, Bayes et al. found that the REMPARK system (comprised of a belt-worn sensor, and smartphone) received a median usability score of 70/100, with 76% of participants “satisfied” or “very satisfied” by the system, but comfort scoring the lowest^44^. Hadley et al. found that 14/16 participants successfully used the KinesiaU for 5-weeks, with 88% agreeing or strongly agreeing that the system was easy to use^72^, but did not report on comfort findings, despite being assessed. Pastorino et al. found that 65% of participants reported that the system (four tri-axial accelerometers placed on each limb) could be used outside of experimental conditions to monitor ON-OFF state, with four reporting that the system was not comfortable^56^. Boroojerdi et al. found that NIMBLE patches were easy to use, not painful to detach, and did not interfere with participant’s daily lives^76^. In Pfister et al.’s study participants rated four statements on a Likert scale, generally observing that the sensor did not bother participants during the activities of daily living and that they would recommend it^73^. Lopane et al. reported that participants completed a three-item, five-level questionnaire to assess the usability of the McRoberts Dynaport, but only reported that overall scores ranged from 3 to 4, with no further information provided^87^. Binder et al. reported that ActiWatch was well tolerated by all participants but did not highlight how this was assessed^75^. Mahadevan et al. found that 85% of participants would be likely or very likely to wear their sensors for an extended period, with 7/8 participants reporting the sternum as the most uncomfortable sensor placement, and wrist placement being the highest^25^.

### Missing Data

Across the included studies, 38/79 papers reported on the presence of missing data (Supplementary Table 4). Two of the 38 studies reported that data was missing, but did not provide any further details^56,89^. An additional, fifteen studies directly reported that all participants had complete datasets (marked as N/A in Supplementary table 4). Whereas 26 studies did not mention whether data sets were complete (marked as UC in Supplementary table 4).

Four overall factors influenced missing data, including protocol deviations, technical issues with WT, participant factors, and lack of medication effect. The most common cause of missing data was protocol deviations (*n* = 17)^26,28,30,31,35,39,44,45,48,63,70,76–78,80,81,83^, specifically related to participants not completing their medication state diaries, the ‘rainy weather’ stopping one participant from performing outside walking, participants not being able to complete walking tests, and missing UPDRS scores. Technical issues with WT were reported in 11 studies^20,23,25,31,40,41,50,52,72–74^ – specifically, due to ‘technical malfunctions’, ‘technical problems with video recording’ so they could not match up with sensor recordings, and problems with Bluetooth connection. Participant related factors were a problem in five studies^22,37,38,47,61,67,68^, this included participants retracting consent, feeling unwell, not wearing their WT, withdrawing from the study, and incorrectly filling in their medication diaries/not completing them. Three studies excluded participants for whom medication effect could not be observed, or they didn’t transition to ON state^29,34,61^.

## Discussion

To our knowledge, this is the first systematic review to provide a comprehensive assessment of studies, which use WT to explore medication effect on motor function and symptoms in PwP. This review highlighted that despite significant technological advances, there is no commonly accepted standard to objectively monitor medication effect in PwP, with great heterogeneity in the technology and protocols used, and the majority of research conducted in controlled laboratory conditions, exploring short-term medication effects.

To overcome the current reliance on subjective and patient-reported (UPDRS) measures of medication effect in PwP, the use of WT is rapidly increasing. However, as observed in this review, this rapid increase has contributed to significant heterogeneity in the protocols used to explore medication effect. For example, amongst the included studies, to explore medication effect, 50 different WTs were placed across 20 locations on the body. This heterogeneity, coupled with the lack of technical/clinical validation of the WT and its outcomes, reduces the ecological and external validity of data collected, with many technologies not utilising open-source algorithms, making it difficult to compare outcomes. The majority of research was also conducted in controlled laboratory or hospital environments, meaning insights into real-world medication effect are still limited. To explore real-world medication effect, future research should prioritise the technical and clinical validation of current WT. For example, the IMI-funded Mobilise-D project have produced open-source highly validated algorithms^97^ to monitor gait in PwP, using validated McRoberts Dynaport MM + . Technology such as this can be used over prolonged time periods in the real-world, and provide highly valid insights into medication effect in PwP. Smartwatches and smartphones^98^ could also be used to monitor medication intake, and individual’s motor function/symptoms in the real-world^99^.

Across the included studies, to monitor specific motor functions/symptoms, WT was placed across multiple locations on the body. For example, to monitor gait, WT was placed across 17 different locations, but most commonly on the wrist. Monitoring gait using wrist-worn sensors reduces the internal validity of findings, as wrist-worn sensors often overestimate physical activity outcomes^100^, and can be influenced by tremor and reduced arm swing^101^ which are common in PwP. Additionally, monitoring gait at various locations means that each study will be monitoring slightly different gait attributes. This highlights the importance of future research exploring the performance of WT based on its location, and then utilising the gold-standard location when monitoring specific motor function/symptoms, such as the feet/shoes or lower back for gait. However, recent feasibility research highlights that wrist-worn sensors are more acceptable to PwP than those placed on the lower back or feet^25,102^. This highlights that when developing or using WT, it is important to consider device usability, feasibility, and acceptance^99^ as they are likely to influence results, especially in individuals with more severe motor symptoms, or those with non-motor symptoms such as cognitive impairment who are most likely to benefit from WT. Usability and feasibility were rarely assessed in the studies included in this review, with usability assessments often limited to tick-box responses and no qualitative feedback. Therefore, in line with the World Health Organisation’s advice, future research should prioritise usability and feasibility analysis when using WT to explore medication effect in PwP.

This review also highlighted that the majority of medication effect studies are performed in controlled, laboratory settings. These environments, often increase attention and motivation, and result in the Hawthorne effect, causing individuals to perform at their optimum^103^. These environments can also induce anxiety, making motor symptoms, such as tremor, more severe^104–106^. Exploring medication effect in the real-world would overcome these limitations and should therefore be prioritised in future research. This real-world research would also enable medication effect to be explored over prolonged time periods (e.g., 7-Days), rather than in a ‘snapshot’ ON-OFF period, as commonly seen in this review (64/79 studies). Additionally, continuous real-world monitoring will allow fluctuating motor function/symptoms to be captured—a factor which is difficult to capture in ON-OFF monitoring.

Although motor symptom fluctuations are difficult to capture in ON-OFF monitoring, ON-OFF research can still provide useful insights into immediate medication effect, and thus has an important place in research. Future research which utilises this method needs to identify the best timings to explore medication effect, as many studies in this review utilised heuristic thresholds, with great heterogeneity in the timings. For example, amongst the 65 studies, which explored ON-OFF medication effect, 22 different timings were used to assess ON state and 16 different timings were used to assess OFF state. As medication effect will differ based on the time since medication intake, these studies will be exploring different factors. For example, Curtze et al. observed no significant change in swing time within 60 min after medication intake^21^, whereas Barbosa et al.^64^ observed a significant change 80 min after intake. This highlights the importance of exploring medication effect over prolonged time periods, and not just an hour after taking medication, which is commonly seen in this review. Furthermore, this highlights the importance of identifying how long after medication intake motor function/symptoms change, thus providing optimum timings to assess ON-OFF medication effect in future research.

Further heterogeneity is introduced by the various protocols used to monitor each motor function/symptom (Supplementary Table 5). For example, to monitor gait, studies used 5-, 7-, 10-, 20-, 30-, 50-m straight and circular walk tests, Timed Up and Go, and unscripted walking in the real-world. Each variation will provide different insights into the medication effect. Future research should focus on real-world, unscripted walking, as this will provide more valid insights.

Parkinsonian medication improved pace-related gait metrics (e.g., gait speed and step length), turning, bradykinesia, and tremor. However, a study by Curtze et al. observed that even the most responsive gait variables were never within a standard deviation of healthy controls, and hence participants motor function and symptoms remained quite impaired following medication intake^21^. Additionally, dopaminergic medication induced dyskinesia and had a negative effect on balance, with limited to no effect on STS variables, dynamic stability metrics, asymmetry, and stride time. Few studies observed a significant effect of levodopa medication on temporal gait variables, with only one of four studies to monitor cadence, observing a significant increase following medication intake. These findings provide support for the potential role of cholinergic activity in gait^107,108^, with support from a study in PwP, observing an increase in gait speed following intake of an acetylcholinesterase inhibitor, which increases the level of acetylcholine^109^.

Interestingly, there were conflicting findings of medication effect on mean gait cycle time, with Curtze et al.^21^ observing a significant decrease in mean gait cycle time following medication intake, but Cebi et al.^22^ observing no significant change. The severe participants in Curtze et al.’s study had the same change in mean gait cycle time following medication, as the participants in Cebi et al.’s study. However, there were only 18 participants in Cebi et al.’s study, in comparison to 104 in Curtze et al.’s study. This highlights the importance of including larger samples, as they may be more powered to observe medication effects. Additionally, Curtze et al. also observed a significant increase in cadence, and it was the only one of four studies, which monitored cadence, to observe this^21^. These findings may reflect the hypothesis that in PwP, to compensate for decreased step velocity and reduced stride length, cadence increases^110^ or stays the same as healthy controls^84^, and therefore cannot be improved by medication. Alternatively, these conflicting findings may be explained by the presence of dyskinesia and FoG^21,84^, or PD severity^21^ which influenced medication effect in various studies. Similarly, this review highlighted that comorbidities, polypharmacy, and interactions between medications may have also influenced medication effect, factors which were not controlled for in most studies included in this review. For example, one study observed that individuals with early PD and mild depression, have more variable and slower gait^4,111^. Therefore, in PwP and depression, the medication effect may be more pronounced. Future research should control for the effects of FoG, dyskinesia, and PD severity.

Across the included studies, medication effect was most commonly assessed on motor function, with fewer studies monitoring motor symptoms. Despite gait being the most commonly assessed motor function, surprisingly few studies explored the effect of medication on gait initiation^21^ and gait variability^95^, factors, which are commonly impaired in PwP. Most research in PwP, which explores medication effect on gait variability is conducted in controlled laboratory environments^112^. Therefore, it is especially important to understand how medication influences gait variability in real-world settings, as lab-based variability measures different factors^113^. As our understanding of medication effect on real-world gait is limited, future research could prioritise this, especially given that the majority of PwP experience gait impairment, meaning these insights would be very beneficial.

Despite all studies monitoring participants before and after medication intake or continuously over a period of time, only 36/79 studies reported the effect of medication in a clinically relevant manner i.e., they reported the strength and direction of medication effect on clinically measurable motor functions and/or symptoms, e.g., reporting the value/change in step length following medication intake. This was a particular problem in studies whose main aim was to identify the accuracy of their algorithms to predict ON-OFF medication state, with these papers generally reporting algorithm accuracy without discussing what the motor functions or symptoms looked like in those states — critical information for clinically relevant insights. Future research should therefore report the values of motor functions/symptoms in both ON-OFF medication states or how the variable changed over time and when that changed occurred, rather than just reporting that gait speed ‘changed’ following medication intake, or that ‘our algorithms could accurately predict ON-OFF states’.

In addition, as Parkinsonian medications have different modes of action and influence different motor functions/symptoms, the specific medication taken by participants should also be reported, a factor which was lacking in many of the included studies, with many simply reporting that ‘medication’ or ‘Parkinsonian’ medication was taken. Furthermore, to allow meta-analysis and summary statistics to be calculated, future research should ensure that the specific medication taken by participants and the LEDD for each participant is reported. Furthermore, 12 studies administered 'suprathreshold' medication doses which may exaggerate the effect of medication on motor function/symptoms in PwP, and therefore are not representative of the true effect.

This review highlighted that there is no standard to objectively monitor medication effect in PwP, with great heterogeneity in the technology and protocols used to assess medication effect. The majority of research is conducted in controlled laboratory conditions, exploring short term medication effects, across a variety of motor functions/symptoms. Key recommendations for future research include exploring medication effect on real-world motor function/symptoms, over prolonged time periods (e.g., 7 days); improving technical and clinical validity of current WT; exploring the feasibility and usability of current WT; and identifying the best times to explore medication effect. Only then will insights enable optimal adaptation of medication regimens for PwP.

## Methods

### Search Method

To provide the most up-to-date review, two searches of 9 databases were run. The first search was run on January 18^th^ 2023, including papers published between January 2000 and January 2023. The second search was run on October 1^st^ 2024, and included papers published January 2023 – October 2024. The nine databases included PubMed, Scopus, Cochrane, IEEE, PsychInfo, Embase, Web of Science, Medline, and CINAHL. The reporting of this systematic review was guided by the Preferred Reporting Items for Systematic Review and Meta-Analysis (PRISMA) Statement^114^ (Supplementary Tables 1 and 2). The search strategy was developed in PubMed using medical subject headings (MeSH) based on the key concepts of “Parkinson”, “medication”, “wearable technologies”, “motor impairment” (Supplementary Table 3). To ensure a comprehensive search, a list of synonyms was identified from the MeSH terms and previous reviews. Duplicates were removed from compiled articles, and the full title and abstracts were screened independently by two reviewers (EP and HD). Any discrepancies during screening and data extraction were resolved by an independent reviewer (SDD). The full text was reviewed for any papers which passed title and abstract screening, in addition to those which were not clear from the title and abstract alone.

### Inclusion and Exclusion Criteria

Studies were included if they monitored motor function and/or symptoms using WT. For the purpose of this review, *motor function* was defined as any behaviour, which requires direct control and coordination of muscles, including gait, balance, sit to stand and turning; and *motor symptoms* were defined as any motor symptom assessed in the MDS-UPDRS; or any Parkinsonian symptom related to impaired motor function (i.e., tremor, dyskinesia, and bradykinesia). Studies were included if they were published in English, if the full text was available, were published in peer-reviewed journal articles, and if the study design was either observational, retrospective, prospective, interventional, randomised control, or a case report.

Studies were excluded if they explored the effect of medication on motor function and symptoms in any disease apart from PD, included animals, performed in-vitro testing, involved cadavers, explored non-motor symptoms (i.e. fatigue and low blood pressure), if participants did not use medication commonly prescribed for Parkinson’s (e.g., medications under clinical trial, or complementary therapies), they did not use WT (e.g., wired technology), and if the effect of medication was not measured (e.g., did not perform assessments before and after medication), if they were conference abstracts, a review, book chapter, clinical trial registration, qualitative study, a comment/letter, only conducted computational modelling (e.g., using simulation data), and if they were written in languages other than English.

### Data Extraction

Using standardised data extraction techniques, data were independently extracted by two reviewers (EP and HD). Key outcomes included study design, PD characteristics (e.g., Hoehn and Yahr Stage, time since diagnosis, LEDD, and MDS-UPDRS III and IV scores), testing protocol, motor function/symptoms assessed, the WT and placement location on the body, study setting (real-world or laboratory/clinic-based), how medication timings were monitored (ON-OFF, or recorded by participant etc), level of compliance and usability of WT, and key findings. Due to a variety of study designs included in this review, a customised quality appraisal (risk of bias) of all included studies was performed. To perform the quality appraisal an adapted version of Downs and Black^115,116^ quality appraisal form for health interventions and the National Institutes of Health (2014) Quality Assessment Tool was used. EP and HD independently conducted a quality appraisal, providing an individual score for each paper. For each paper, EP and HD gave a score of 1 (Yes) or 0 (No or Unclear) for 15 questions (e.g., was the study population clearly defined?). The two scores were then averaged, providing an overall score for each paper (e.g., reviewer 1 = 10, and reviewer 2 = 9, this paper would score 9.5) (Supplementary Table 4). Papers were scored out of 15, with scores ≥ 13 rated Excellent, Good = 9–12; Moderate 5–8; Poor = 0–4.

## Supplementary information

Supplementary Material

## Data Availability

The datasets used and/or analysed during the current study is available from the corresponding author on reasonable request.
