# Supplementary material for: Systematic review of wearables assessing medication effect on motor function and symptoms in Parkinson’s disease
Source: NPJ Parkinsons Dis. 2025 May 22;11:135. doi: 10.1038/s41531-025-00943-y (PMC12098882; doi:10.1038/s41531-025-00943-y)
Supplement: Supplementary file 1 — Supplementary Material [file 41531_2025_943_MOESM1_ESM.docx]

|  | **Was the study population clearly specified and defined? (Age/gender/condition)** | **Were inclusion and exclusion criteria for participants defined? (mark no if determined after study onset)** | **Was the sampling method described? (e.g. random, consecutive, opportunity)** | **Were participants representative of the entire population they were recruited from?** | **Was missing data or attrition addressed with clearly defined reasons?** | **Was the research question or objective in this paper clearly stated?** | **Are the main outcomes to be measured clearly described in the Methods/Introduction. Could it be replicated?** | **Validated measures (criterion/convergent/discriminant validity) and implemented consistently across all study participants?** | **Appropriate ethics and consent?** | **Is the sample size justified with a power analysis and/or a study referencing a similar sample size?** | **Were the statistical tests used to assess the main outcomes appropriate (i.e. parametric vs. non-parametric)?** | **Probability values reported (e.g. 0.026 rather than <0.05) for the main outcomes. Note - unless less than 0.001** | **Were key potential confounding variables measured and adjusted statistically for their impact on the outcome(s)?** | **Aims of study fully met and clearly described?** | **Was reporting of results adequate (i.e. no selective reporting)** | **Quality Assessment - Poor = 0-4 Moderate = 5-8 Good = 9-12 Excellent = ≥13** | **Quality Assessment - Poor = 0-4 Moderate = 5-8 Good = 9-12 Excellent = ≥13** | **Average** |
| --- | --- | --- | --- | --- | --- | --- | --- | --- | --- | --- | --- | --- | --- | --- | --- | --- | --- | --- |
| **Author** |  |  |  |  |  |  |  |  |  |  |  |  |  |  |  | **Rater 1** | **Rater 2** |  |
| **Aghanavesi S et al ^59^** | YES | NO | NO | NO | UC/YES | YES | YES | YES | YES | NO | YES | YES | NO | YES | YES | 9 | 10 | **9.5** |
| **Agurto C et al ^20^** | YES | YES | NO | NO | YES | YES | YES | YES/UC | YES | NO | YES | YES | NO | YES | YES | 11 | 10 | **10.5** |
| **Aich S et al ^57^** | YES | YES | NO | NO | N/A | YES | YES | YES | YES | NO | YES | NO | NO | YES | YES | 10 | 10 | **10** |
| **Antonini A et al ^63^** | YES | NO | NO | YES/UC | YES | YES | YES | YES | YES | NO | YES | YES | NO | YES | NO/YES | 11 | 10 | **10.5** |
| **Atrsaei A et al ^30^** | YES | YES | NO | NO | YES | YES | YES | YES | YES | NO | NO | YES | NO | YES | YES | 10 | 10 | **10** |
| **Barbosa R et al ^64^** | YES | YES | YES | YES/UC | UC/YES | YES | YES | YES | YES | NO | YES | NO | NO | YES | YES | 11 | 11 | **11** |
| **Bayes A et al ^44^** | YES | YES | YES | NO | YES | YES | YES | YES | YES | NO | YES | NO | NO | YES | NO | 10 | 10 | **10** |
| **Beck et al ^95^** | YES | YES | NO | UC | UC | YES | YES | YES | YES | NO | YES | YES | YES | YES | YES | 11 | 11 | **11** |
| **Binder et al ^75^** | YES | YES | NO | NO | UC / N/A | YES | YES | YES | YES | NO | YES | NO | NO | YES | YES | 9 | 10 | **9.5** |
| **Boroojerdi B et al ^76^** | YES | YES | NO | NO | YES | YES | YES | YES | YES | YES | UC/NO | NO | NO | YES | YES | 10 | 9 | **9.5** |
| **Borzi L et al ^66^** | YES | NO | NO | UC | UC / N/A | YES | YES | YES | YES | NO | UC/YES | YES | NO | YES | YES | 8 | 10 | **9** |
| **Cai G et al ^79^** | YES | NO | NO | UC | UC / N/A | YES | NO | UC | YES | NO | YES | YES | NO | YES | YES | 7 | 8 | **7.5** |
| **Carissimo C et al ^89^** | YES | YES | YES | YES/UC | NO | YES | YES | YES | YES | NO | YES | YES | YES | YES | YES | 13 | 11 | **12** |
| **Cebi I et al ^22^** | NO | YES | YES | YES | YES | YES | YES | YES | YES | NO | YES | YES | NO | YES | YES | 12 | 12 | **12** |
| **Channa et al ^42^** | NO | NO | NO | UC | UC / N/A | YES | YES | YES | NO/UC | NO | YES | YES | NO | YES | YES | 7 | 8 | **7.5** |
| **Chatzaki C et al ^50^** | YES | NO | NO | NO | YES | YES | YES | YES | YES | NO | YES | YES | YES | YES | YES | 11 | 11 | **11** |
| **Corra MF et al ^78^** | YES | YES | NO | YES | YES | YES | YES | YES | YES | YES/NO | YES | YES | NO | YES | YES | 13 | 12 | **12.5** |
| **Curtze C et al ^21^** | YES | YES | NO | UC | N/A | YES | YES | YES | YES | NO | YES | YES | YES | YES | YES | 12 | 12 | **12** |
| **Czech M et al ^23^** | YES | YES | NO | UC | YES | YES | YES | YES | YES | NO | YES | YES | NO | YES | YES | 11 | 11 | **11** |
| **Di Lazzaro et al ^67^** | YES | NO | YES | YES | YES / N/A | YES | YES | YES | YES | NO | YES | YES | NO | YES | NO | 12 | 11 | **11.5** |
| **Elshehabi M et al ^29^** | YES | NO | YES | YES/UC | YES | YES | YES/NO | YES | YES | NO | YES | YES | YES | YES | YES | 13 | 11 | **12** |
| **Evers et al ^31^** | YES | YES | NO | NO | YES | YES | YES | YES | YES | NO | YES | YES | YES | YES | YES | 12 | 12 | **12** |
| **Fernandes et al ^32^** | NO | YES | YES | YES | UC / N/A | YES | YES | YES | YES | NO | YES | NO | YES | YES | YES | 11 | 12 | **11.5** |
| **Ferreira et al ^33^** | YES | YES | YES | YES | UC / N/A | YES | YES | YES | YES | NO | YES | YES | YES | YES | YES | 12 | 13 | **12.5** |
| **Fisher et al ^80^** | NO | YES | NO | UC | YES | YES | YES | YES | YES | NO | YES | YES | YES | YES | YES | 11 | 11 | **11** |
| **Ghoraani et al ^54^** | YES | YES | NO | NO | N/A | YES | YES | UC | YES | NO | YES | YES | NO | YES | YES | 11 | 11 | **11** |
| **Griffiths et al ^36^** | NO | NO | NO | NO | N/A | YES | YES | YES | YES | NO | UC | NO | NO | YES | NO | 6 | 6 | **6** |
| **Grigoriou et al ^47^** | YES | YES | NO | YES/UC | YES | YES | YES | UC | YES | NO | YES | YES | NO/YES | YES | YES | 11 | 11 | **11** |
| **Habets et al ^34^** | YES | YES | NO | NO | YES / N/A | YES | YES | YES | YES | NO | UC/NO | YES | NO | YES | YES | 11 | 9 | **10** |
| **Hadley et al ^72^** | YES | NO | NO | UC | YES | YES | YES | YES | YES | NO | UC | NO | NO | YES | YES | 8 | 8 | **8** |
| **Horak et al ^24^** | NO | YES | NO | UC | UC / N/A | YES | YES | YES | YES | NO | NO/YES | YES | YES | YES | YES/NO | 11 | 10 | **10.5** |
| **Hssayeni et al ^58^** | YES | NO | NO | NO | UC | YES | YES | UC | NO | NO | YES | YES | YES | YES | YES | 8 | 8 | **8** |
| **Hssayeni et al ^60^** | YES | NO | NO | NO | UC | YES | YES | YES | YES | NO | YES | YES | YES | YES | YES | 10 | 10 | **10** |
| **Hssayeni et al ^61^** | YES | YES | NO | NO | YES | YES | YES | YES | YES | NO | YES | NO | YES | YES | YES | 11 | 11 | **11** |
| **Hua et al ^51^** | YES | NO | NO | NO | UC | YES | YES | YES | YES | NO | YES | YES | YES | YES | YES | 10 | 10 | **10** |
| **Iijima et al ^88^** | YES | NO | NO | YES | N/A | YES | YES | UC | YES | NO | UC | YES | NO | YES | YES | 9 | 9 | **9** |
| **Johansson D et al ^43^** | YES | NO | NO | UC | UC | YES | YES | YES | YES | NO | YES | YES | NO | YES | YES | 9 | 9 | **9** |
| **Keijsers et al ^68^** | YES | YES | NO | NO | YES / UC | YES | YES | UC | YES | NO | YES | YES | NO | YES | YES | 11 | 10 | **10.5** |
| **Khodakarami et al ^37^** | NO | NO | NO | UC | YES | YES | YES | YES | YES | NO | YES | YES | NO | YES | YES | 9 | 9 | **9** |
| **Knudson et al ^38^** | YES | NO | NO | UC | YES | YES | YES | YES | YES | NO | UC | YES | NO | YES | YES | 9 | 9 | **9** |
| **Lebel et al ^69^** | YES | NO | NO | NO | UC | YES | YES | YES | YES | NO | YES | YES | NO | YES | YES | 9 | 9 | **9** |
| **Li W et al ^48^** | YES | NO | NO | YES | NO | YES | YES | YES | NO | NO | YES | YES | NO/YES | YES | NO/YES | 9 | 10 | **9.5** |
| **Lohle M et al ^74^** | YES | YES | NO/YES | YES/UC | YES | YES | YES | YES | YES | NO | YES | YES | NO/YES | YES | YES | 12 | 13 | **12.5** |
| **Lonini et al ^70^** | YES | NO | NO | NO | YES | YES | YES | UC | YES | NO | UC/NO | YES | NO | YES | YES | 8 | 8 | **8** |
| **Lopane et al ^87^** | YES | YES | NO | NO | UC | YES | YES | YES | YES | NO | YES | YES | YES | YES | YES | 11 | 11 | **11** |
| **Mahadevan et al ^25^** | YES | YES | NO | NO | YES | YES | YES | YES | YES | NO | YES | YES | NO | YES | YES | 11 | 11 | **11** |
| **Marin et al ^53^** | YES | YES | NO | UC | UC | YES | YES | YES | YES | NO | YES | YES | NO | YES | YES | 10 | 10 | **10** |
| **Mera et al ^81^** | YES | NO | NO | NO | YES | YES | YES | YES | YES | NO | YES | NO | NO | YES | YES | 9 | 9 | **9** |
| **Mileti et al ^55^** | YES | YES | NO | UC | N/A | YES | YES | YES | YES | NO | YES | NO | NO | YES | YES | 10 | 10 | **10** |
| **Miller Koop et al ^49^** | YES | YES | NO | UC | UC | YES | YES | YES | YES | NO | YES | YES | NO | YES | YES | 10 | 10 | **10** |
| **Moore et al ^90^** | NO | NO | NO | NO | N/A | YES | YES | YES | YES | NO | YES | NO | YES | YES | NO | 8 | 8 | **8** |
| **Moradi et al ^77^** | NO | NO | NO | UC | YES | YES | YES | YES | NO | NO | NO | YES | NO | YES | YES | 7 | 7 | **7** |
| **Myers et al ^86^** | NO | NO | NO | NO | N/A | YES | YES | UC | NO | NO | UC | NO | NO | YES | YES | 5 | 5 | **5** |
| **Nguyen et al ^45^** | NO | YES | NO | YES | YES | YES | YES | UC | NO | NO | YES | YES | YES | YES | YES | 10 | 10 | **10** |
| **Ohara M et al ^65^** | YES | YES | YES/NO | YES/UC | UC | YES | YES | UC | YES | NO | YES | YES | NO | YES | YES | 11 | 9 | **10** |
| **Pastorino et al ^56^** | NO | YES | NO | NO | NO | YES | NO | UC | NO | NO | N/A | NO | NO | YES | NO | 4 | 4 | **4** |
| **Pedrosa et al ^83^** | YES | NO | NO | UC | YES | YES | YES | UC | YES | NO | YES | YES | YES | YES | YES | 10 | 10 | **10** |
| **Perez-Lopez et al ^93^** | NO | NO | NO | NO | UC | YES | YES | YES | YES | NO | N/A | YES | NO | YES | YES | 8 | 8 | **8** |
| **Perez-Lopez et al ^96^** | YES | YES | NO | NO | UC | YES | YES | YES | YES | NO | N/A | YES | NO | YES | YES | 10 | 10 | **10** |
| **Pfister et al ^73^** | YES | NO | NO | UC | YES | YES | YES | YES | YES | NO | UC | YES | NO | YES | YES | 9 | 9 | **9** |
| **Rahimi et al ^62^** | YES | YES | NO | YES | N/A | YES | YES | YES | YES | NO | YES | NO | YES | YES | YES | 12 | 12 | **12** |
| **Ramesh et al ^26^** | NO | NO | NO | UC | YES | YES | YES | YES | YES | NO | YES | YES | NO | YES | YES | 9 | 9 | **9** |
| **Raykov et al ^35^** | NO | YES | NO | UC | YES | YES | YES | YES | NO | NO | YES | YES | NO | YES | YES | 9 | 9 | **9** |
| **Ricci et al ^16^** | YES | YES | NO | UC | UC | YES | YES | YES | YES | NO | YES | YES | NO | YES | YES | 10 | 10 | **10** |
| **Rosqvist et al ^39^** | YES | YES | NO | YES | YES | YES | YES | YES | YES | NO | YES | YES | NO | YES | YES | 12 | 12 | **12** |
| **Sadhu S et al ^52^** | YES | NO | NO | NO | YES | YES | YES | YES | NO | NO | YES | YES | NO/YES | YES | YES | 9 | 10 | **9.5** |
| **Sama et al ^92^** | NO | NO | NO | NO | UC | YES | YES | YES | YES | NO | YES | YES | NO | YES | YES | 8 | 8 | **8** |
| **Sayeed et al ^91^** | NO | NO | NO | NO | UC | YES | YES | UC | NO | NO | NO | YES | NO | YES | YES | 5 | 5 | **5** |
| **Sherrill et al ^71^** | NO | NO | NO | NO | UC | YES | YES | UC | NO | NO | YES | NO | YES | YES | NO | 5 | 5 | **5** |
| **Sturman et al ^15^** | YES | NO | NO | NO | N/A | YES | YES | UC | YES | NO | YES | NO | NO | YES | YES | 8 | 8 | **8** |
| **Suppa et al ^84^** | YES | YES | NO | UC | UC | YES | YES | YES | YES | NO | YES | YES | NO | YES | YES | 10 | 10 | **10** |
| **Thomas et al ^82^** | YES | NO | NO | UC | YES | YES | YES | YES | YES | NO | YES | YES | YES | YES | YES | 11 | 11 | **11** |
| **Thomas I et al ^40^** | YES | NO | NO | UC | YES | YES | YES | YES | YES | NO | UC | YES | NO | YES | YES | 9 | 9 | **9** |
| **Tosi et al ^27^** | YES | YES | NO | NO | N/A | YES | YES | YES | NO | NO | YES | YES | NO | YES | NO | 9 | 9 | **9** |
| **Watts et al ^41^** | YES | YES | NO | UC | YES | YES | YES | YES | YES | NO | YES | NO | NO | YES | YES | 10 | 10 | **10** |
| **Weiss et al ^85^** | YES | YES | NO | UC | UC / N/A | YES | YES | YES | YES | NO | YES | YES | YES | YES | YES | 11 | 12 | **11.5** |
| **Wilken et al ^94^** | YES | YES | YES | YES | UC / N/A | YES | YES | UC | YES | NO | YES | YES | YES | YES | YES | 12 | 13 | **12.5** |
| **Wu X et al ^28^** | YES | YES | YES | YES | YES | YES | YES/NO | YES | YES | YES/NO | YES | YES | NO/YES | YES | YES | 14 | 13 | **13.5** |
| **Zhang et al ^46^** | YES | NO | NO | UC | N/A | YES | YES | YES | YES | NO | YES | NO | NO | YES | YES | 9 | 9 | **9** |
